# Application of the metagenomic next-generation sequencing in diagnosis and therapy of chronic kidney disease patients with infections

**DOI:** 10.12669/pjms.40.4.9244

**Published:** 2024

**Authors:** Dongrui Liang, Xiaodong Li

**Affiliations:** 1 Dongrui Liang, Department of Ophthalmology, Baoding No. 1 Central Hospital of Hebei Medical University, Baoding 071000, Hebei, China; 2 Xiaodong Li, Department of Nephrology, Baoding No. 1 Central Hospital of Hebei Medical University, Baoding 071000, Hebei, China

Chronic Kidney Disease (CKD) and end-stage renal disease are significant public health issues that are prevalent in the 21st century. Patients with impaired kidney function experience notable immune dysregulation compared to the general population. Immune dysfunction in CKD patients occurs regardless of the underlying disease and presents early in the course of renal insufficiency. Infections are a major contributor to illness and death among patients at all stages of CKD, ranking as the second most common cause of mortality and increasing the risk for cardiovascular events in CKD patients, leading to frequent hospitalizations. Studies have demonstrated that both the naive and acquired immune systems are compromised in these individuals, with substantial consequences on morbidity and mortality.^1^

Metagenomic next generation sequencing (mNGS) is an emerging method with the potential for screening pathogens, offering the ability to significantly improve diagnostic accuracy when conventional culture methods yield negative results.^2^ mNGS involves the rapid sequencing of nucleic acid sequences in samples using a second-generation sequencing platform, which are then compared with genomic sequences of individual species to identify and quantify microorganisms present. The process of detecting pathogenic microorganisms using mNGS typically involves six steps: sample collection from the patient’s infection sites, extraction of nucleic acids, construction of a standard sequencing library, high-throughput sequencing, bioanalysis to identify pathogenic bacteria, and interpretation of the results.

mNGS has the advantages of providing comprehensive coverage of pathogenic microorganisms in the tested samples, being time-efficient, and unbiased. There have been numerous research reports on the application of mNGS technology to assist in the diagnosis of infectious diseases, including infections in almost all parts of the body such as the respiratory system, central nervous system, urinary system, digestive system, musculoskeletal system, skin, eyes, and blood.^3^ Particularly in cases of complex infections in immunocompromised patients, where traditional tests struggle to identify the pathogen or rare pathogen infections, mNGS technology has shown unique advantages in clinical diagnosis.

mNGS offers significant benefits for diagnosing a variety of conditions, including urinary tract infections, peritoneal dialysis-related peritonitis, hemodialysis catheter-related bloodstream infections and lung infections in patients with CKD.^4^ This technology is particularly effective for identifying fungi, atypical pathogens, parasites, and mixed infections. By accurately detecting pathogens, mNGS enables the implementation of targeted treatments, thereby reducing unnecessary and inappropriate antibiotic use and preventing clinical antibiotic misuse. Moreover, mNGS is more efficient and less time-consuming than traditional culture methods.

While mNGS offers unique advantages in clinical microbiology, there are still challenges to address, such as its ability to detect certain difficult, undetectable, and low-burden microorganisms, as well as the impact of different extraction methods used by various laboratories on mNGS results. Additionally, the broad coverage of microorganisms presents a challenge in interpreting mNGS results, requiring technicians to consider the pathogenicity and conditions of different microorganisms and establish individualized reporting thresholds, while clinicians need extensive experience and specialized expertise. Furthermore, the high cost of mNGS currently limits its widespread clinical application.

In conclusion, mNGS has been utilized for diagnosing infections in the bloodstream, nervous system, and respiratory tract by identifying pathogens in diverse samples including blood, cerebrospinal fluid, and alveolar lavage fluid specimens. Research has shown that the mNGS approach can identify a wide range of pathogens such as bacteria, fungi, viruses, and parasites, offering benefits such as improved sensitivity, increased throughput, reduced testing time, and decreased dependence on patient conditions.^5^ Hence, it has promising prospects for pathogen diagnosis and is worthy of further exploration.

## Authors’ contributions:

**XL:** Design and revision.

**DL:** Preparation of manuscript and literature review.

All authors contributed to the article and approved the submitted version.
